# Elucidation of in Vitro Chlorinated Tyrosine Adducts in Blood Plasma as Selective Biomarkers of Chlorine Exposure

**DOI:** 10.1021/acs.chemrestox.2c00053

**Published:** 2022-05-27

**Authors:** Mirjam de Bruin-Hoegée, Irene M. van Damme, Tomas van Groningen, Debora van der Riet-van Oeveren, Daan Noort, Arian C. van Asten

**Affiliations:** † van ‘t Hoff Institute for Molecular Sciences, Faculty of Science, University of Amsterdam, P.O. Box 94157, Amsterdam 1090GD, The Netherlands; ‡ TNO Defence, Safety and Security, Dep. CBRN Protection, Lange Kleiweg 137, Rijswijk 2288GJ, The Netherlands; § CLHC, Amsterdam Center for Forensic Science and Medicine, University of Amsterdam, P.O. Box 94157, Amsterdam 1090GD, The Netherlands

## Abstract

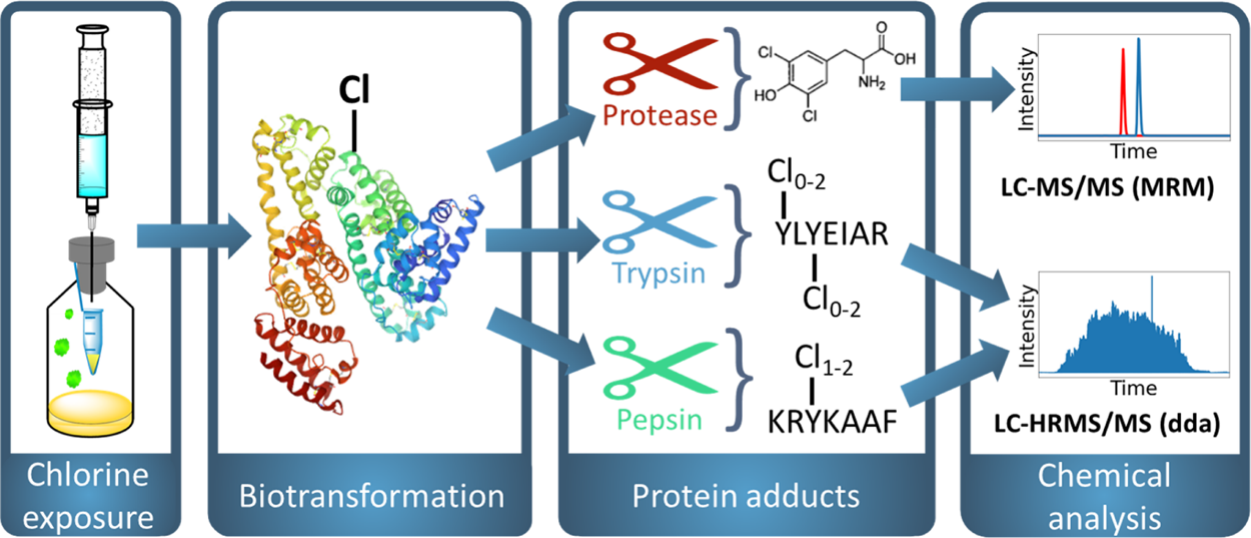

Chlorine is a widely available industrial chemical and involved in a substantial number of cases of poisoning. It has also been used as a chemical warfare agent in military conflicts. To enable forensic verification, the persistent biomarkers 3-chlorotyrosine and 3,5-dichlorotyrosine in biomedical samples could be detected. An important shortfall of these biomarkers, however, is the relatively high incidence of elevated levels of chlorinated tyrosine residues in individuals with inflammatory diseases who have not been exposed to chlorine. Therefore, more reliable biomarkers are necessary to distinguish between endogenous formation and exogeneous exposure. The present study aims to develop a novel diagnostic tool for identifying site-specific chlorinated peptides as a more unambiguous indicator of exogeneous chlorine exposure. Human blood plasma was exposed in vitro to various chlorine concentrations, and the plasma proteins were subsequently digested by pronase, trypsin, or pepsin. After sample preparation, the digests were analyzed by liquid chromatography tandem mass spectrometry (LC–MS/MS) and liquid chromatography high-resolution tandem mass spectrometry (LC–HRMS/MS). In line with other studies, low levels of 3-chlorotyrosine and 3,5-dichlorotyrosine were found in blank plasma samples in this study. Therefore, 50 site-specific biomarkers were identified, which could be used as more unambiguous biomarkers for chlorine exposure. Chlorination of the peptides TY*ETTLEK, Y*KPGQTVK, Y*QQKPGQAPR, HY*EGSTVPEK, and Y*LY*EIAR could already be detected at moderate in vitro chlorine exposure levels. In addition, the latter two peptides were found to have dichlorinated fragments. Especially, Y*LY*EIAR, with a distinct chlorination pattern in the MS spectra, could potentially be used to differentiate exogeneous exposure from endogenous causes as other studies reported that this part of human serum albumin is nitrated rather than chlorinated under physiological conditions. In conclusion, trypsin digestion combined with high-resolution MS analysis of chlorinated peptides could constitute a valuable technique for the forensic verification of exposure to chlorine.

## 1 Introduction

Chlorine (Cl_2_), a highly reactive and toxic gas, is one of the most abundantly used industrial chemicals, with an annual production of multi-million tons.^1,2^ It has a wide variety of industrial applications, including the production of polymers and chlorinated solvents, separation of metals in mining, the disinfection of drinking water, and use within the bleaching industry.^3^ It has been the cause of a significant number of cases of acute poisoning.^4,5^ Although known as a toxic dual-use chemical, it has not been scheduled by the Organization for the Prohibition of Chemical Weapons (OPCW), mainly because the large scale of production and storage makes verification or inspections practically impossible.^6^ Notwithstanding, its use as a weapon is an obvious violation of the Chemical Weapons Convention. Chlorine gas was the first deployed chemical warfare agent during World War I, resulting in many victims.^7^ Recently, the OPCW published multiple reports stating that chlorine has been used in the ongoing conflict in the Syrian Arab Republic.^8−13^

Exposure to chlorine can cause severe acute and long-term health effects. Inhalation exposure to 1–3 ppm is already associated with mild irritation of mucous membranes.^4^ Based on this value, the recommended exposure limit is 0.5 ppm for longer-term exposure and the acceptable short-term exposure limit is 1 ppm.^14^ Eye and throat irritation will develop at exposure levels between 5 and 15 ppm.^4^ Levels exceeding 15 ppm result in cough, chest pain, and choking.^15^ From a concentration of 50 ppm, damage to the main airways and acute pulmonary edema occur.^2^ In general, a dose above 400 ppm is lethal as this high concentration will result in respiratory arrest, hemorrhage, and acute burns of the upper and proximal lower airways.^15^ Long-term exposure can result in similar symptoms as short-term exposure below 50 ppm, where in 10% of the cases, incomplete recovery after symptomatic treatment was reported.^16^

Especially in the case of major incidents with many affected individuals, methods for rapid triage and diagnosis of chlorine exposure are indispensable. However, detection of intact agents in biomedical samples is often not possible due to the reactivity of chemical threat agents. Furthermore, other traces of evidence of intentional release are usually difficult to obtain as well. For this purpose, biomarkers of exposure are used for verification purposes. Metabolic indicators for chlorine poisoning are the phospholipid l-α-phosphatidylglycerol,^17^ chlorinated lipids such as 2-chloropalmitaldehyde and 2-chlorosearaldehyde,^18^ 8-isoprostane (8-isoPGF_2α_) as a marker of lipid peroxidation,^19^ and adducts to tyrosine, such as 3-chlorotyrosine (Cl-Tyr) and 3,5-dichlorotyrosine (di-Cl-Tyr).^20−23^ In particular, tyrosine chlorination yields persistent biomarkers that can be found days after chlorine poisoning.^21^

**Figure 1:**
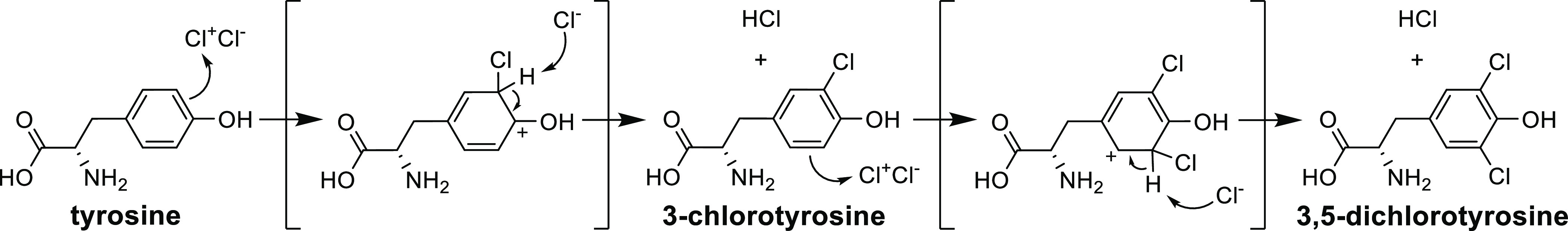
Reaction scheme of electrophilic aromatic substitution of tyrosine by chlorine, leading to the formation of 3-chlorotyrosine and 3,5-dichlorotyrosine.

During chlorine gas exposure, chlorine reacts in the body with aqueous mucus on epithelial tissues to form hydrochloric acid and hypochlorite.^2^ Protein adducts can be formed when chlorine oxidizes tyrosine by electrophilic aromatic substitution.^21^ The aromatic ring in tyrosine is particularly reactive due to its electron-donating hydroxyl group. Figure 1 shows the reaction of tyrosine with chlorine, where substitution is directed toward the ortho position, leading to the formation of Cl-Tyr and di-Cl-Tyr.^24^

An important shortfall of these biomarkers, however, is the relatively high incidence of elevated levels of chlorinated tyrosine residues in individuals who have not been exposed to chlorine. Chlorinated tyrosine biomarkers were found in people with inflammatory diseases,^21,25,41^ diabetes mellitus,^26^ and atherosclerotic vascular disease.^27^ For instance, Buss et al. reported that infants with respiratory distress had more than 6 times higher Cl-Tyr levels in tracheal aspirate proteins detected by GC–MS, compared to the control group without known diseases.^25^ In addition, for patients with inflammatory disease, maximum levels of 20 and 5 ng/mL blood have been analyzed by liquid chromatography tandem mass spectrometry (LC–MS/MS) for Cl-Tyr and di-Cl-Tyr, respectively.^21^ Interestingly, in the referred study, both the healthy and diseased groups showed a relatively high Cl-Tyr level with a large variation, which makes verification even more difficult. In this respect, the di-Cl-Tyr adduct has been considered slightly more specific for exogenous chlorine exposure than the Cl-Tyr adduct.^21,28^ Nonetheless, verification of a chlorine attack based on these biomarkers is less reliable because of the potential presence of Cl-Tyr and di-Cl-Tyr in the blood plasma of non-exposed individuals. Therefore, more unambiguous biomarkers of chlorine exposure are necessary to differentiate exogeneous exposure from endogenous processes.

A powerful tool to obtain sequence information for individual peptides is the use of liquid chromatography–high-resolution tandem mass spectrometry (LC–HRMS/MS) for bottom-up proteomics. Data-dependent analysis in combination with database searching can be used to screen for peptides with post-translational modifications (PTMs). This strategy has successfully led to the identification of biomarkers for the exposure to other chemical threat agents.^29,30^ The validity of such an approach within the context of Cl-Tyr being a biomarker of inflammatory diseases has recently been reported by Nybo et al. in various publications.^31−33^ Detailed analysis of site-specific peptide modifications is expected to lead to improved differentiation of endogenous reactions due to oxidative stress and exogeneous chlorine exposure because the former will cause both chlorination and nitration of tyrosine, whereas the latter will only result in chlorination.^34,35^

Consequently, the current study aims to develop a diagnostic tool for identifying chlorine–tyrosine biomarkers as robust and specific indicators of exogeneous chlorine exposure. First, human blood plasma was exposed in vitro to various chlorine concentrations. Subsequently, the isolated plasma proteins were digested by pronase, trypsin, or pepsin and analyzed by LC–MS/MS and LC–HRMS/MS. The data were processed by Peaks X+ software and manually interpreted to identify several chlorinated peptides of interest. The present work shows that such specific chlorinated peptides are indeed formed and, therefore, might serve as promising biomarkers for verification of human exposure to chlorine.

## 2 Experimental Procedures

### 2.1 Safety

Due to the potent nature of chlorine gas, all experiments were performed in a fume hood by trained personnel. Precautions were taken to prevent accidental exposure, including the use of gloves and eye protection.

### 2.2 Chemicals

Acetic acid, ABC, calcium hypochlorite, 3-chloro-l-tyrosine, dithiothreitol (DTT), formic acid, pepsin from porcine gastric mucosa (≥2,500 units/mg protein), protease from *Streptomyces griseus* (pronase, ≥3.5 units/mg solid), sodium acetate trihydrate, sodium iodoacetate, trifluoroacetic acid, trypsin from the bovine pancreas (≥10 000 BAEE units/mg protein), and urea were obtained from Sigma-Aldrich (Zwijndrecht, The Netherlands). Acetonitrile, acetone, and methanol were purchased from Biosolve (Valkenswaard, The Netherlands). A peptide mixture of bovine serum albumin protein digest (BSA, LOT #UH285651) and hydrochloric acid were obtained from ThermoFisher Scientific (Landsmeer, The Netherlands). Additionally, water (MilliQ, SimPak 1), 3,5-dichloro-l-tyrosine (BOC Sciences, London, UK), and ^13^C_6_-3-chloro-l-tyrosine (Cambridge Isotopic Laboratories, Andover MA, USA) were employed. The purities of the agents were higher than 97%. Human plasma was purchased from Sanquin (Amsterdam, The Netherlands).

### 2.3 In Vitro Chlorine Exposure

**Figure 2:**
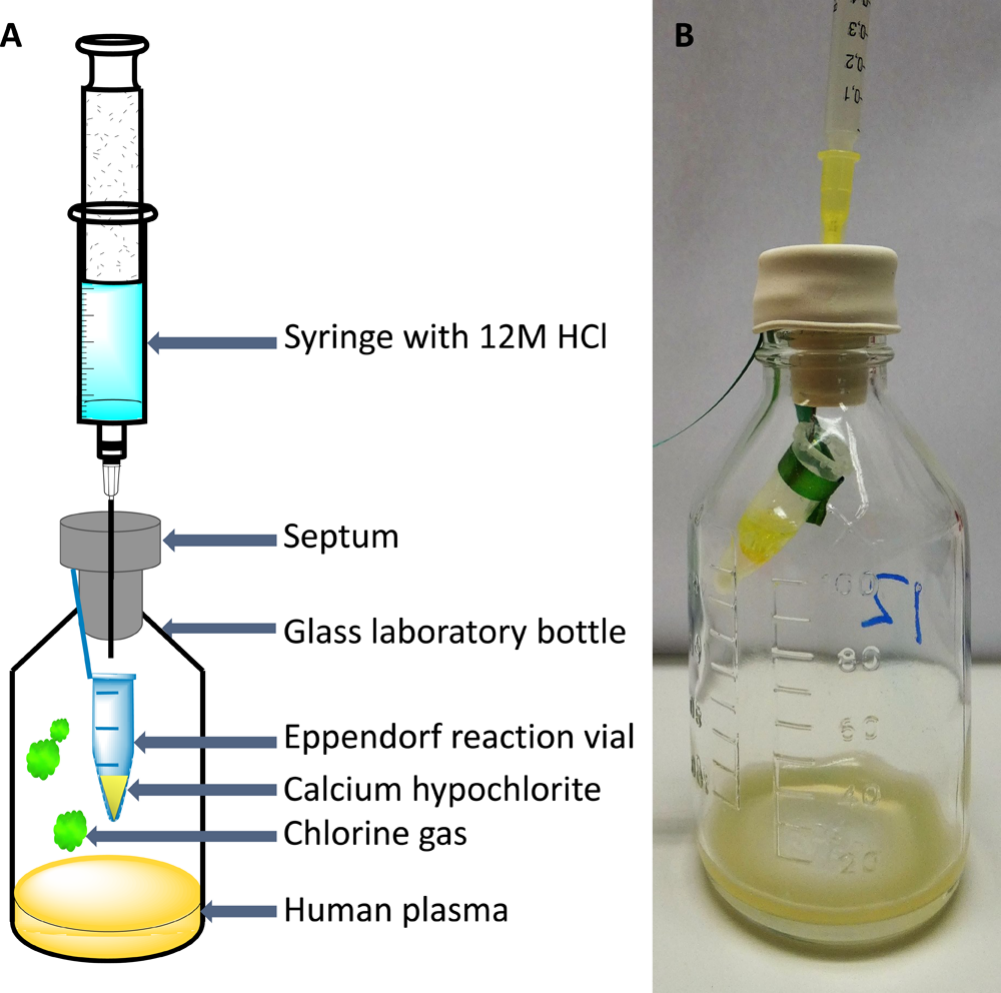
Experimental setup for in vitro exposure of human plasma to in situ generated chlorine gas. (A) Schematic view. (B) Photo of the exposure system.

The experimental setup is visualized in Figure 2. Human plasma (2 mL) was transferred to a 100 mL glass laboratory bottle. A 1.5 mL Eppendorf tube with 1, 10, and 50 mg of calcium hypochlorite was placed in the bottle above the plasma sample. The reaction vial was sealed with a rubber septum and parafilm. A syringe was put through the septum, and slowly, over a time period of 1 min, 1 mL of a 12 M HCl solution was added to the calcium hypochlorite, after which 10, 100, or 500 ppm chlorine gas was generated in situ for 2 h. These concentrations will be described as low, medium, and high concentrations because the chlorine uptake in the plasma, the extent of adduct formation, and the comparison between in vitro and in vivo exposure are unknown. Also, negative controls, that is, blanks, were included without HCl and calcium hypochlorite. The experiments were performed in triplicate for each concentration including the blanks.

### 2.4 Protein Precipitation and Digestion

After chlorine exposure, all protein was precipitated by addition of 10 mL of acetone, followed by centrifugation in a 15 mL Corning tube at 2000 rpm for 4 min (Heraeus Megafuge 1.0R). The acetone layer was discarded, and the steps were repeated. Afterward, the protein precipitate was allowed to dry in the air at ambient temperature.

After precipitation, the isolated protein was digested by pronase, trypsin, or pepsin. Pronase digests the protein up to individual amino acids, while trypsin only cleaves arginine (R) and lysine (K), resulting in longer peptides.^36^ Pepsin is a nonspecific protease with phenylalanine (F), lysine (K), arginine (R), and proline (P) as favored residues.^37^ Pepsin and trypsin allow analysis of specific chlorinated peptides. For pronase digestion, 3 mg of isolated protein was dissolved in 500 μL of aqueous ammonium bicarbonate (ABC, 50 mM) and an aqueous solution of 100 μL of pronase (10 mg/mL in 50 mM ABC). The samples were incubated overnight in a Thermoshaker (Grant-bio PHMT) at 37 °C and 800 rpm.

In addition, the protein was digested by trypsin. First, isolated protein was dissolved in 1 mL of urea (8 M solution in 50 mM ABC), containing 5 μL of DTT (800 mM in water). The solution was incubated for 45 min at 37 °C and 800 rpm. After incubation, 100 μL of sodium iodoacetate (150 mM in water) was added for carboxymethylation of reduced cysteine residues to prevent reformation of disulfide bonds. Subsequently, the solution was incubated for 30 min in the dark at 37 °C and 800 rpm. Afterward, the sample was filtered through a 10 kDa Amicon ultra-centrifugal filter at 14 000 rpm for 10 min in an Eppendorf centrifuge (5417R). The residue was washed on the filter four times with 400 μL of ABC buffer (50 mM) and then collected and dissolved in 400 μL of water. Then, 30 μL of trypsin solution (10 mg/mL in 50 mM acetate buffer, pH = 3.55) was added to the dissolved residue. The sample was incubated overnight at 37 °C and 800 rpm.

In addition, the isolated protein was dissolved in 300 μL of pepsin solution (62.5 μg/mL formic acid). The solution was incubated for 1.5 h at 37 °C and 800 rpm.

### 2.5 Sample Preparation

After pronase digestion, the sample was filtered through a 3 kDa Amicon ultra-centrifugal filter at 14 000 rpm for 10 min in an Eppendorf centrifuge. The filtrate was transferred to an LC–MS vial and analyzed as described in Section 2.6.

After trypsin and pepsin digestion, the samples were filtrated through a 10 kDa filter at 14 000 rpm for 10 min. To avoid pollution of the LC-Orbitrap-MS, the filtrates were purified with reversed-phase solid-phase extraction (SPE) using a C18 column (Bakerbond SPE). To wet the sorbent bed and activate the nonpolar sorbents, 1 mL of methanol was percolated through the column and 1 mL of water was used to equilibrate the column. Afterward, 100 μL of the filtrate was loaded onto the sorbent bed and washed with 1 mL of water to desalt the peptides and remove other hydrophilic compounds from the sample matrix. The retained analytes were eluted with 1 mL of 60% acetonitrile in water. Afterward, the sample was dried under nitrogen and dissolved in 1 mL of water with 1% v/v formic acid. The final elute was transferred to an LC–MS vial and analyzed as described in section 2.6.

### 2.6 Chemical Analysis

#### 2.6.1 LC–MS/MS Selected Reaction Monitoring

The pronase digests were diluted 10- to 1000-fold with water depending on the concentration prior to analysis on a Waters Acquity ultra-high pressure liquid chromatographic (UPLC) system equipped with a Waters Acquity HSS T3 C18 column (100 × 2.1 mm I.D., 1.8 μm). The blanks were not further diluted, except by the addition of the internal standard, resulting in a 1.1-fold dilution. The mobile phase consisted of water (Eluent A) and acetonitrile (Eluent B), both with 0.2% formic acid, using a gradient at a flow of 100 μL/min. Gradient elution started at 100% eluent A for 1 min, followed by linear ramping to 80% eluent B in 8 min and maintaining this composition for 2 min. After each analysis, the system was equilibrated at 100% eluent A for 3 min. The injection volume was 5 μL at a temperature of 8 °C. The analysis was performed at room temperature. The UPLC system was coupled to a Waters (Milford, MA, USA) Xevo TQ-S triple-quadrupole mass spectrometer, equipped with electrospray ionization (ESI), for quantification of the analytes in the positive ionization mode. The capillary voltage was set to 3.5 kV with a nitrogen cone gas flow of 150 L/h and a cone voltage of 10 V. The collision gas argon was set at a flow of 0.19 mL/min. Data were acquired with the selected reaction monitoring mode. The monitored transitions were *m*/*z* 216.2 → *m*/*z* 170.3 at a collision energy (CE) of 15 eV and *m*/*z* 216.2 → *m*/*z* 135.3 (CE = 25 eV) for Cl-Tyr, *m*/*z* 250.1 → *m*/*z* 204.0 (CE = 25 eV) and *m*/*z* 250.1 → *m*/*z* 169.0 (CE = 30 eV) for di-Cl-Tyr, and *m*/*z* 222.0 → *m*/*z* 176.3 (CE = 15 eV) and *m*/*z* 222.0 → *m*/*z* 141.3 (CE = 25 eV) for the internal standard ^13^C_6_-3-chloro-l-tyrosine.

#### 2.6.2 LC-HRMS/MS Data-Dependent Acquisition

The trypsin and pepsin digests were analyzed on an LC-HRMS/MS instrument consisting of an Ultimate 3000 RSLCnano system (Thermo Scientific Dionex Softron GmbH, Germany) coupled to an Orbitrap mass spectrometer (Q Exactive plus, Thermo Scientific, Bremen, Germany). First, 10 μL of the sample was injected onto an Acclaim PepMap 100 C18 μ-precolumn (5 mm × 300 μm I.D., 5 μm, 100 Å, Thermo Fisher Scientific) of 30 °C and washed with the loading solvent (0.05% trifluoroacetic acid in water) at a flow of 30 μL/min for 3 min. Subsequently, the peptides were separated on an Acclaim PepMap C18 Analytical column (250 mm × 75 μm I.D., 2 μm, 100 Å, Thermo Fisher Scientific) at a constant flow of 300 nL/min at a temperature of 35 °C. Gradient elution started at 98% eluent A (0.1% v/v formic acid in water), followed by a linear increase to 25% eluent B (0.1% v/v formic acid in acetonitrile) in 10 min. After the analysis, eluent B was further increased to 80% in 5 min, and this composition was maintained for 5 min. The column was returned to the initial conditions in 5 min and equilibrated for 23 min. The nanoLC system was coupled to the mass spectrometer using an EASY-spray source. Positive ESI analysis was performed with a spray voltage of 1.5 kV. The ion-transfer capillary temperature was set at 250 °C. A full-scan range of *m*/*z* 300–2000 was applied at a resolution of 70 000. Subsequently, data-dependent acquisition was performed. Of each spectrum, the 10 most abundant ions were selected for MS/MS analysis with a positive charge of 2–4 and a resolution of 17 500. The performance of the system was checked by measuring a quality control sample of a peptide mixture of 100 fmol BSA digest, where at least a coverage of 60% was required.

### 2.7 Data Analysis

The raw LC-HRMS/MS data were analyzed using Peaks X+ software (2019, Bioinformatics Solutions Inc., Waterloo, Canada). First, a de novo search was performed, which calculates all theoretically possible peptides. Afterward, a PEAKS DB Search was employed, which compared all these peptides with the FASTA database, limiting the results to peptide sequences that were found in the database. The de novo algorithm interprets the tandem mass (MS/MS) spectrum of each peptide by calculating the mass differences between fragment ions and assigning these mass differences to specific amino acid residues. Three types of variable PTMs were selected: single chlorination, dichlorination, and nitration of tyrosine. The maximum allowed variable PTM per peptide was set to 8, and the maximum missed cleavages was set to 6. The error tolerances for the precursor mass and the fragment ion were set at 0.1 and 0.5 Da monoisotopic mass, respectively. Each amino acid in the de novo sequence was assigned a local confidence score. Default settings were used, while removing peptide sequences with an average local confidence score below 50%. From each peptide, all amino acids with a local confidence higher than 30% were included in the de novo sequence tag of the peptides. This tag was used for the PEAKS DB search. The resulting peptides were exported and statistically analyzed by the open-source software Python 3.9.5 using difflib as part of the standard python package. The code written for this research is published under a GNU General Public License.^38^ Because spectral interpretation was based on mass only, excluding the isotope pattern that is clearly discernible for chlorine, the MS/MS spectra of the most relevant peptides were also interpreted manually. Duplicate compounds, peptides with an area smaller than 10^4^ a.u., and peptides of nonhuman origin were removed. In addition, only peptides that were present in both the blank and highly exposed samples and with enhanced chlorination in the latter were selected for further research. The percentage of chlorinated tyrosine adducts (PoC) was calculated using eq 1
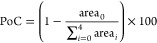
1where area_0_ is the response of the unchlorinated peptide and  is the sum of the responses of the single, double, triple, and quadrupole chlorinated peptides, detected by LC-HRMS/MS. The total number of observed chlorine atoms for each peptide did not exceed 4. This calculation is based on the assumption of a uniform MS response between native and chlorinated species of a specific peptide.

## 3 Results and Discussion

### 3.1 Targeted LC–MS/MS Analysis of 3-Chlorotyrosine and 3,5-Dichlorotyrosine

Before presenting the results of site-specific biomarkers, the overall Cl-Tyr and di-Cl-Tyr levels were established in a similar manner as reported by Crow et al.^21^ The results of the optimization and validation of the corresponding targeted LC–MS/MS method can be found in Section S1 of the Supporting Information. These levels can be used to indicate for which chlorinated tyrosine concentrations, site-specific peptides will be found, as explained in Section 3.2. In addition, this section will clarify that these tyrosine biomarkers cannot always be used to distinguish victims exposed to low chlorine levels from individuals who have not been exposed to chlorine at all.

**Figure 3:**
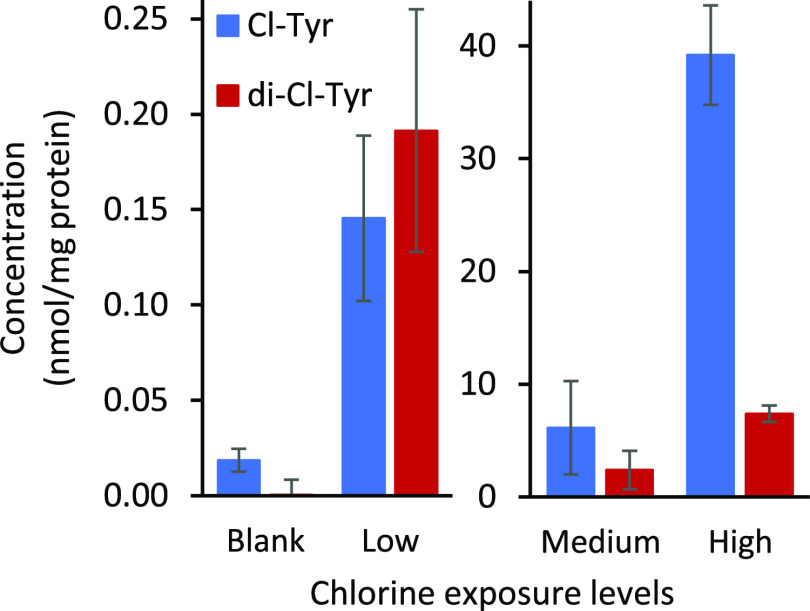
Effect of various chlorine gas exposure levels on 3-chlorotyrosine (Cl-Tyr) and 3,5-dichlorotyrosine (di-Cl-Tyr) concentrations. Error bars represent ± 1SD (standard deviation) for *n* = 3.

Figure 3 shows the detected concentrations of Cl-Tyr and di-Cl-Tyr for the various chlorine exposure levels. The measured concentrations for the lowest exposure level were 0.15 ± 0.04 and 0.19 ± 0.06 nmol/mg protein for Cl-Tyr and di-Cl-Tyr, respectively. The concentration of Cl-Tyr in this sample was only 8 times higher than the concentration in the blank nonexposed sample. The Cl-Tyr values in the blank were above the limit of quantification (LOQ) of 0.9 pmol/mg protein and could easily be distinguished. The di-Cl-Tyr concentration in the blank was much lower and was consequently just below the LOQ of 0.7 pmol/mg protein. However, it had a signal-to-noise (S/N) ratio of at least 6 and the ion ratio deviated less than 6% from the reference standard, and it could therefore be identified.^39^ The overall ratio between Cl-Tyr/Tyr detected by LC-HRMS/MS as depicted in Tables S1 and S2 (Supporting Information) shows a similar tendency as the concentrations analyzed by LC–MS/MS, although the error range of the LC-HRMS/MS results was much larger, resulting in an overlap between medium and blank concentrations.

To evaluate how representative these chlorotyrosine levels are for concentrations found in the blood of exposed victims, the detected concentrations were compared to values reported in the literature. Nishio et al. examined the Cl-Tyr levels in an autopsy sample of a person who possibly died of chlorine and drug poisoning and reported a Cl-Tyr concentration of 60 ng/mL in left heart blood, analyzed by GC–MS.^22^ This is a lower concentration than observed for the blank and the lowest exposure level in this study (0.15 and 1.2 μg/mL blood, respectively) assuming that blood consists of 55% plasma and the amount of protein in human plasma is 70 mg/mL, as determined by the Bradford assay.^40^ Nevertheless, many factors might influence the detected concentration, such as the moment of sampling after death, preservation of the samples, and matrix effects. The Cl-Tyr levels that are analyzed by LC–MS/MS in rat and mouse models of chlorine exposure causing labored breathing and severe lung injury^18^ are slightly higher than the levels found by Nishio and are 10 times lower than the concentrations observed at the lowest exposure level in this study.

These results show that chlorinated tyrosine levels in an exposed victim are not substantially higher than these values in healthy individuals. The difference is probably even smaller for diseased people with elevated chlorinated tyrosine levels. Being aware of the limitations of the chlorinated tyrosine adduct method, this study aims to identify more unambiguous biomarkers of chlorine exposure (Section 3.2.) that can be more specifically linked to inhalation of chlorine gas.

### 3.2 Data-Dependent LC-HRMS/MS Analysis of Chlorinated Peptides

#### 3.2.1 Identification of Chlorinated Peptides

**Figure 4:**
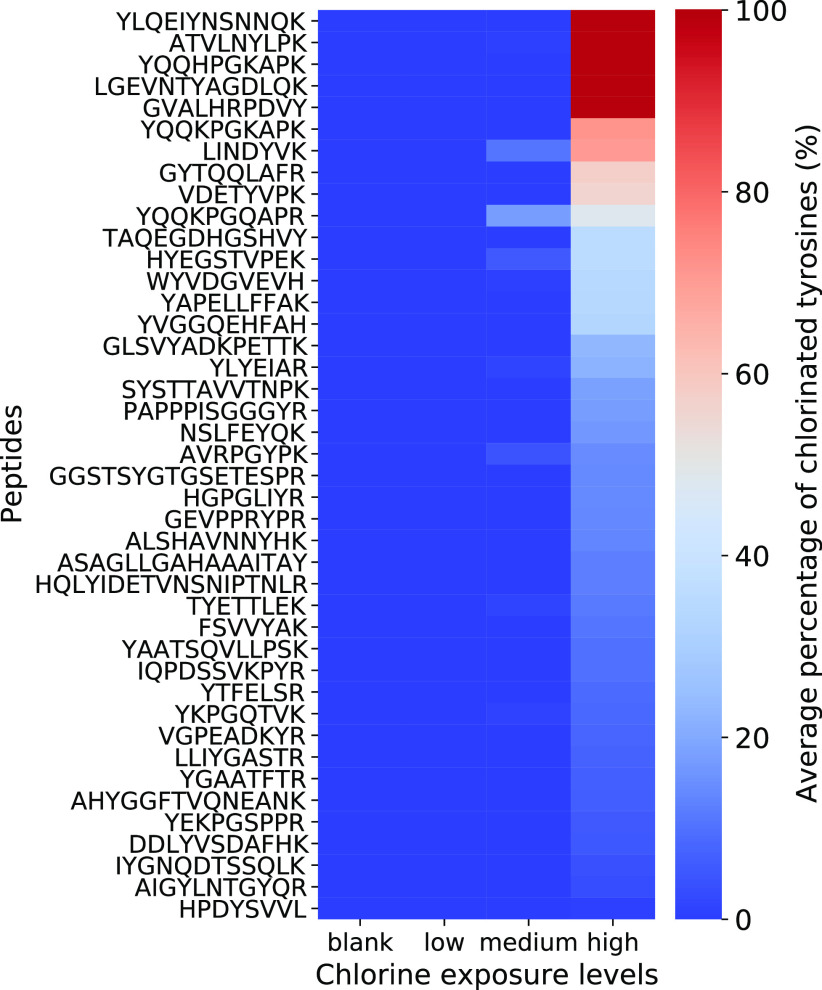
Heatmap of peptides identified with LC-HRMS/MS after trypsin digestion of human blood plasma, with the corresponding average percentage of chlorinated tyrosines (PoC) for no exposure and low, medium, and high chlorine exposure.

In this section, the results will be described from analyzing peptide fragments containing chlorinated tyrosine residues, rather than focusing on the overall chlorinated tyrosine amino acid levels after pronase digestion. In the trypsin digests, 42 peptides were identified with LC-HRMS/MS, which could be used as potential biomarkers for chlorine exposure. Figure 4 shows peptides with the average percentage of chlorinated tyrosines at various exposure levels. A clear increase in chlorinated peptides with increasing chlorine exposure concentration is visible. Furthermore, only a limited number of peptides showed significant chlorination at medium chlorine exposure as chlorination of most peptides is only observed at the highest chlorine concentration. A vast majority of the peptides show either no modification or a single chlorination. Double chlorination was only observed for the peptides YQQKPGKAPK, YLYEIAR, and HYEGSTVPEK. The same trends are visible for the eight peptides that were identified as potential biomarkers after pepsin digestion (Figure S2 and Table S1, Supporting Information). An extensive overview of the identified biomarkers with the corresponding mass and retention time can be found in Table S3 of the Supporting Information. Two chlorinated biomarkers were detected in both the trypsin and pepsin digests, and these were chlorinated at the same tyrosine amino acid position. Additionally, not all biomarkers were detected in all repetitions at the highest concentration. This might be due to variation in the sample preparation efficacy and chlorine uptake of the plasma sample. The combination of various digestion methods enabled the identification of more peptides than with a single digestion method, and consequently a larger part of the protein sequence was covered. The coverage of human serum album and haptoglobin was 56 and 33%, respectively (Figure S4, Supporting Information). It should be noted that a few chlorinated peptides were detected in the blank and low exposure levels and not in higher chlorine exposure levels, although the blood plasma of the same donor was used. This unexpected finding was not investigated further as these peptides were not considered to be suitable markers for chlorine exposure.

Table 1 lists the most promising chlorinated peptides as biomarkers for exogeneous chlorine exposure. Because trypsin can cleave the protein on various amino acids and the efficiency is not 100%, the length of some chlorinated peptides varied slightly. The additional amino acids that were occasionally found are indicated with a dot notation of the chlorinated peptide sequence. All peptides were found to be unchlorinated in the blank samples and chlorinated at high chlorine exposure levels. The chlorinated variants of the peptides HY*EGSTVPEK, TY*ETTLEK, Y*KPGQTVK, Y*LY*EIAR, and Y*QQKPGQAPR were detected in the medium chlorine exposure samples as well. This indicates that these peptides might even be used in case a lower concentration of chlorine exposure occurs. Two of these peptides were found to have a single and double chlorinated tyrosine: HY*EGSTVPEK with either one or two chlorines on the tyrosine and Y*LY*EIAR with one to three chlorines attached, where the second tyrosine could be dichlorinated. This might be a region of, respectively, the haptoglobin or albumin protein which is more readily accessible to chlorine.

**Table 1:** Overview of Peptides Containing at Least One Chlorinated Tyrosine Residue, Present in the Precipitated Proteins from Human Plasma Exposed to Various Chlorine Concentrations

blank peptide	chlorinated peptide	protein	accession
HYEGSTVPEK	HY(Cl)EGSTVPEK.K	haptoglobin	P00738|HPT_HUMAN
	HY(Cl_2_)EGSTVPEK.K	haptoglobin	P00738|HPT_HUMAN
TYETTLEK	TY(Cl)ETTLEK	albumin	P02768|ALBU_HUMAN
YKPGQTVK	SI.Y(Cl)KPGQTVK	alpha-2-macroglobulin	P01023|A2MG_HUMAN
YLYEIAR	KK.Y(Cl)LYEIAR	albumin	P02768|ALBU_HUMAN
	K.Y(Cl)LY(Cl)EIAR	albumin	P02768|ALBU_HUMAN
	Y(Cl)LY(Cl_2_)EIAR	albumin	P02768|ALBU_HUMAN
YQQKPGQAPR	Y(Cl)QQKPGQAPR	immunoglobulin kappa variable 3–20	P01619|KV320_HUMAN

Interestingly, other studies have indicated that the peptide Y*LY*EIAR can become (mono and di) nitrated but not chlorinated under physiological conditions.^35,42^ Additionally, LAK*TYETTLEK has also been reported as a biomarker for retrospective detection of human exposure to the nerve agent tabun, with K* as the site of modification.^43^

The Peaks X+ software facilitated rapid analysis and evaluation of the many MS/MS spectra. Furthermore, interpreting each MS/MS spectrum according to the same fixed set of rules reduces human bias, which is important from a forensic point of view. Nevertheless, both the automated analysis and its results require critical human expert evaluation. Error tolerance thresholds for the parent and fragment ion masses turned out to be of significant influence on the identified peptides and the modifications. This is essential because only mass differences are used for identification of the chlorine PTMs, while other relevant information, such as the isotope pattern, is not incorporated as this can hamper protein identification rates.^44,45^ In addition, the isomers of Y(Cl)LYEIAR and Y(Cl)LY(Cl_2_)EIAR were only found manually due to their trace level and the applied concentration threshold settings.

In future research, it would be interesting to examine potential differences between short-term exposure to a high concentration of chlorine gas and long-term low-level exposure. Because of the persistence of blood protein adducts, it is conceivable that the chlorination is cumulative, which may result in a higher concentration of chlorinated biomarkers for low-level long-term exposure than is expected based on a single exposure. Hence, the degree of monochlorination versus dichlorination and the chlorination-to-nitration ratio of tyrosine residues might be useful to investigate exposure conditions.^21,35^

Additionally, it should be emphasized that the in vitro chlorine gas exposure setup represents a simplified model in relation to actual human exposure and does not account for chemical and biological interactions that take place in the various parts of the human body. It is still under debate whether Cl_2_ is fully converted to HOCl and HCl in wet tissues before it reacts with biological molecules or that it will predominantly react with biological compounds before it can undergo hydrolysis. A comprehensive theoretical review by Squadrito et al. suggests that direct reaction of Cl_2_ with biological molecules is kinetically favored.^46^ However, other authors state, based on in vitro experiments, that hypochlorous acid (HOCl) reacts with amino acids and triggers an inflammatory response in the lungs as a result of chlorine exposure.^2,47,48^ For this reason, verification of the chlorinated peptide biomarkers in samples of chlorine-exposed victims is required to establish their value for forensic practice.

#### 3.2.2 Mass Spectrometric Approach for Assigning Chlorinated Peptides

**Figure 5:**
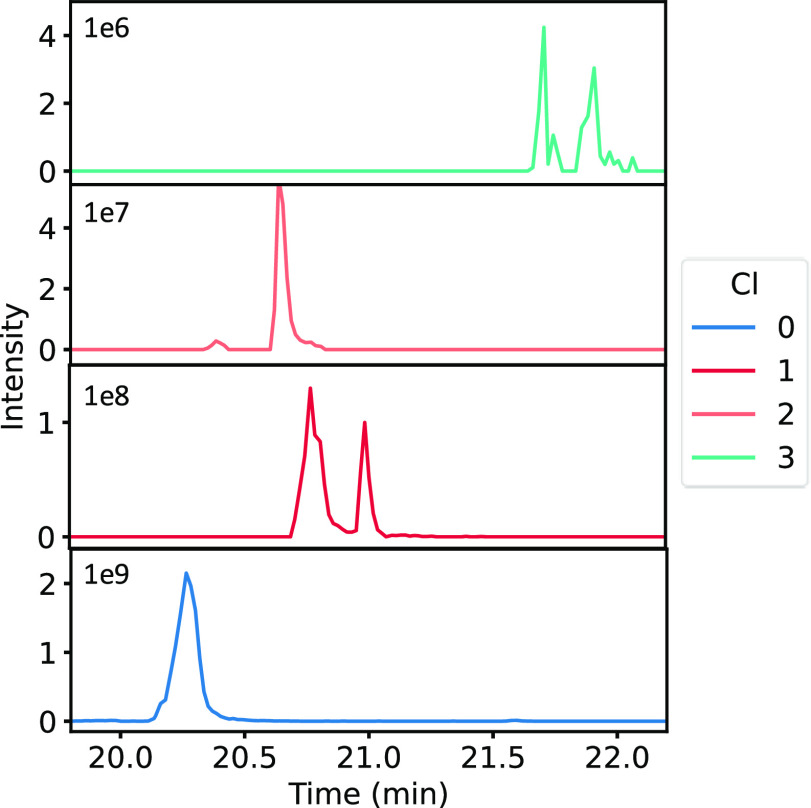
EICs of plasma exposed to the high chlorine exposure concentration analyzed by LC-HRMS/MS, with YLYEIAR at *m*/*z* 464.25 and *t*_R_ = 20.1–20.7 min (blue), Y(Cl)LYEIAR and YLY(Cl)EIAR at *m*/*z* 481.23 and *t*_R_ = 20.8–21.0 and 21.0–21.2 min (red), K.Y(Cl)LY(Cl)EIAR at *m*/*z* 562.26 and *t*_R_ = 20.6–20.8 min (orange), and Y(Cl)LY(Cl_2_)EIAR and Y(Cl_2_)LY(Cl)EIAR at *m*/*z* 515.19 and *t*_R_ = 21.7–21.9 and 21.9–22.0 min (green).

**Figure 6:**
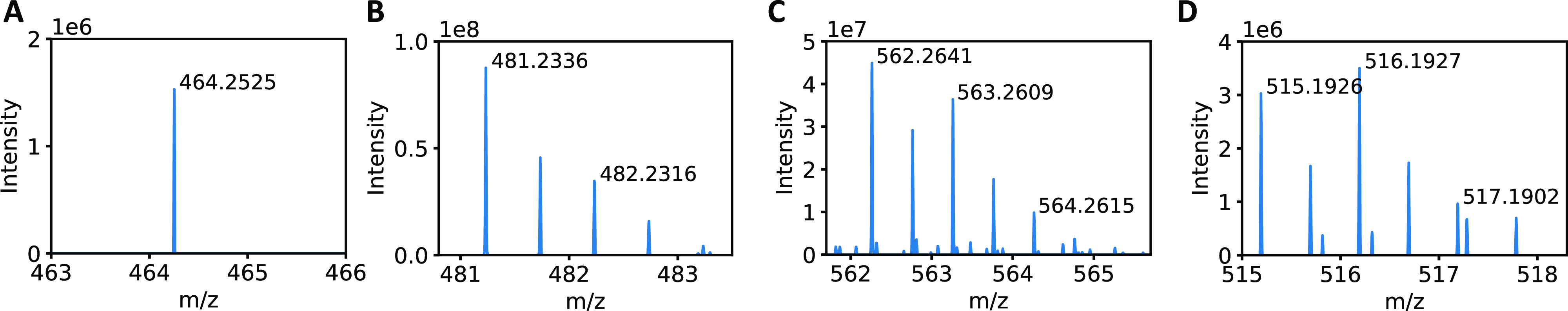
Full scan MS spectrum analyzed by LC-HRMS/MS of doubly charged chlorinated precursor Y*LY*EIAR showing the chlorine isotope pattern: (A) no chlorination, (B) single chlorination with an isotope ratio of around 2:1, (C) double chlorination of K.Y*LY*EIAR with an isotope ratio of 5:4:1, and D) triple chlorination with an isotope ratio of 11:12:5:1.

**Figure 7:**
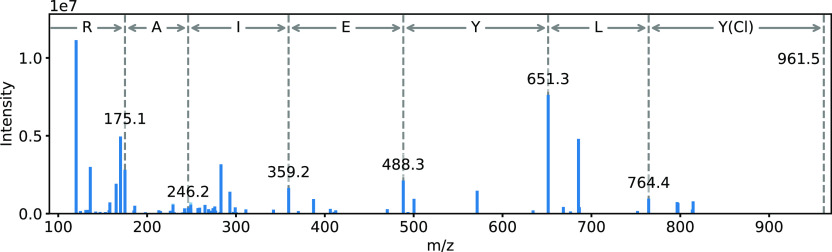
MS/MS spectrum of the parent ion Y(Cl)LYEIAR with an *m*/*z* 481.233 at a t_R_ of 20.76 min, present in the precipitated proteins from human plasma exposed to medium and high concentrations of chlorine gas. The *m*/*z* of the y-fragments and the corresponding amino acids are shown.

The following section discusses the LC-HRMS/MS analysis of two of the chlorinated peptides, that is, Y*LY*EIAR and HY*EGSTVPEK, in more detail. Figure 5 shows the extracted ion chromatograms (EICs) of the mono-, di-, and trichlorinated peptide Y*LY*EIAR in plasma exposed to the highest chlorine concentration, with the corresponding retention times (*t*_R_). The results for the blanks and plasma exposed to other concentrations are shown in the Supporting Information (Figure S4). No chlorinated peptides were detected in the blank. Two peaks are visible at *m*/*z* 481.23 (one chlorine atom) and *m*/*z* 515.19 (three chlorine atoms) because either the first or the second tyrosine can exhibit (di)chlorination. In the full scan MS spectrum of this doubly charged peptide, a distinct chlorine pattern is visible for single, double, and triple chlorination (Figure 6B–D). The unchlorinated peptide showed a single peak as expected (Figure 6A). Table S4 in the Supporting Information demonstrates the excellent correlation of the measured versus theoretical isotope ratios for the acquired data.^49^Figure 7 shows the MS/MS spectrum of the monochlorinated peptide Y(Cl)LYEIAR. All y-ions were found, and their *m*/*z* ions are shown in the spectrum. The highest *m*/*z* 961.5 corresponds to the protonated ion [M + H^+^] of the peptide. The corresponding y-ions and the theoretical b-ions can be found in Table S5 of the Supporting Information. The MS/MS spectra and the corresponding fragmentation pattern of the di- and trichlorinated peptide YLYEIAR are shown in Figures S5–S6 and Tables S6 and S7 of the Supporting Information.

**Figure 8:**
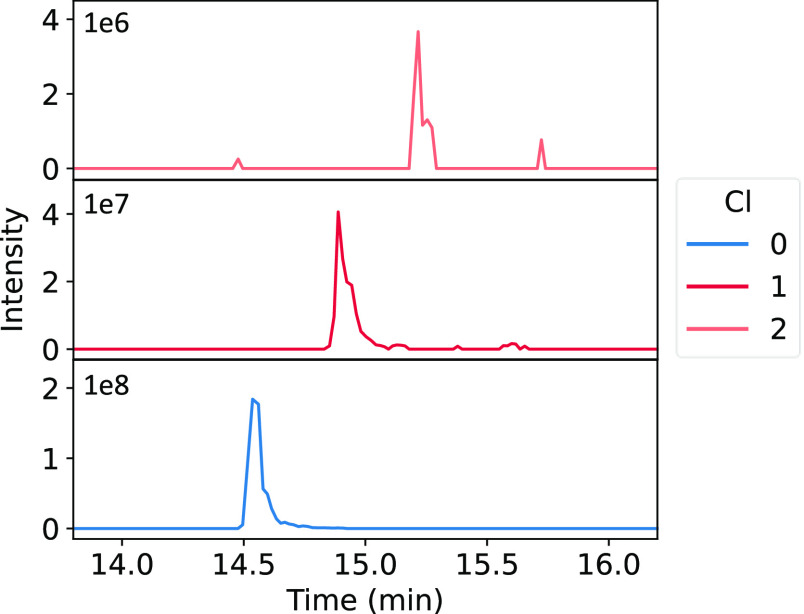
EICs of plasma exposed to the highest chlorine concentration analyzed by LC-HRMS/MS, with HYEGSTVPEKK at *m*/*z* 637.823 and *t*_R_ = 14.3–15.0 min (blue), HY(Cl)EGSTVPEKK at *m*/*z* 654.805 and *t*_R_ = 14.9–15.0 min (red), and HY(Cl_2_)EGSTVPEKK at *m*/*z* 671.785 and *t*_R_ = 15.2–15.3 min (orange).

**Figure 9:**
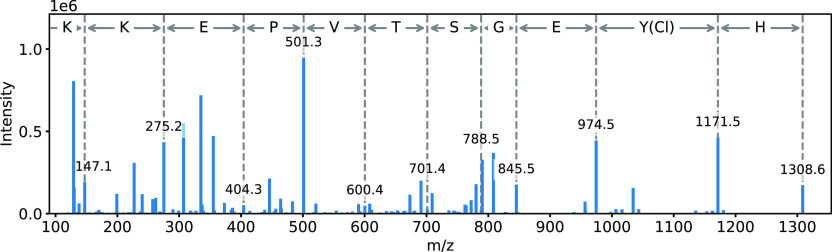
MS/MS spectrum of the parent ion HY(Cl)EGSTVPEKK with an *m*/*z* 654.804 at an *t*_R_ of 14.88 min, detected in the trypsin digest of a plasma sample exposed to medium and high concentrations of chlorine gas. The *m*/*z* of the y-fragments and the corresponding amino acids are shown.

The monochlorinated peptide HY*EGSTVPEKK showed the highest intensity compared to all chlorinated peptides in both the medium and highly exposed samples and is present as an unchlorinated peptide in the blank. Because of its relevance, the EICs of the mono- and dichlorinated peptide HY*EGSTVPEKK in plasma exposed to the highest chlorine concentration are shown in Figure 8. The results for the blank samples and plasma exposed to other concentrations are presented in the Supporting Information (Figure S7). The MS/MS spectrum corresponding to HY(Cl)EGSTVPEKK is shown in Figure 9. All y-fragments and the [M + H^+^] precursor ion were found and depicted in Table S8 in the Supporting Information. Also, all except one of the b-fragments could be identified in the spectrum. The MS/MS spectrum and the corresponding fragmentation pattern of the dichlorinated peptide HY*EGSTVPEKK is shown in Figure S8 and Table S9 of the Supporting Information.

#### 3.2.3 Chlorinated Sites in Human Serum Albumin

**Figure 10:**
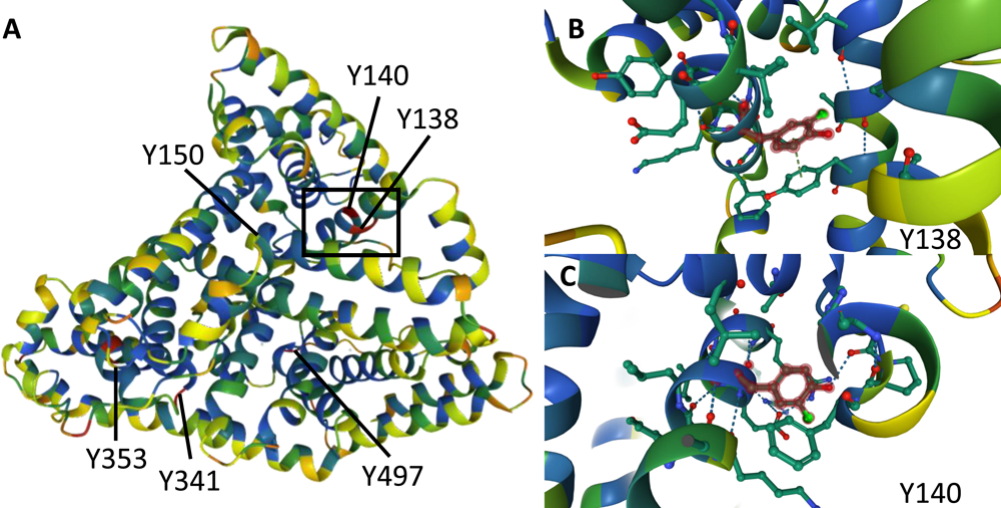
(A) Positions of chlorinated tyrosine residues in human serum albumin. (B) Chlorinated tyrosine residue Y138 of the peptide sequence Y(Cl)LYEIAR. (C) Chlorinated tyrosine residue Y140 of the peptide sequence YLY(Cl)EIAR. The figure was created using uniprot.org © 2002–2022 UniProt Consortium.

To better understand the protein structure and interactions, the positions of the chlorinated tyrosine residues in human serum albumin are visualized in Figure 10. The locations of the identified chlorinated peptides are marked in Figure 10A. Figure 10B,C shows a magnification of the Y138 and Y140 sites of the single chlorinated peptides Y(Cl)LYEIAR and YLY(Cl)EIAR, respectively.

## 4 Conclusions

In the current study, 50 chlorinated peptides have been identified after in vitro exposure of human plasma to chlorine. These peptides could potentially serve as biomarkers to verify exogeneous chlorine exposure in humans. Within this set, five chlorinated peptides were considered to be especially promising biomarkers due to their consistent presence in chlorine exposure samples, the occurrence of multiple degrees of chlorination, and the presence of the unchlorinated peptide in the blank. These peptides, HYEGSTVPEK, TYETTLEK, YKPGQTVK, YLYEIAR, and YQQKPGQAPR, have been detected in the medium exposure concentration samples as well and can be valuable when considering actual samples of potential chlorine exposure victims. Since most of these peptides are derived from proteins that are abundantly present in whole blood, forensic scientists might be able to assess alleged chlorine exposures in a relatively straightforward way. Because the current study works with a simplified in vitro exposure system, subsequent validation of these biomarkers in authentic biomedical samples is, however, pivotal. Nonetheless, the developed method allows for robust and specific analysis of chlorinated adducts formed in blood. Such biomarkers might be used to discriminate between endogenous processes and exogeneous exposure, which is particularly relevant for forensic cases with multiple plausible explanations or in which suspected parties strongly deny the use of chlorine gas as a chemical warfare agent.

## Supporting Information Available

Method optimization and validation, MS/MS spectra, protein coverage, and extensive list of identified biomarkers (PDF)
