# Supplementary material for: Elucidation of in Vitro Chlorinated Tyrosine Adducts in Blood Plasma as Selective Biomarkers of Chlorine Exposure
Source: Chem Res Toxicol. 2022 May 27;35(6):1070–9. doi: 10.1021/acs.chemrestox.2c00053 (PMC9214762; doi:10.1021/acs.chemrestox.2c00053)
Supplement: Supplementary file 1 — tx2c00053_si_001.pdf [file tx2c00053_si_001.pdf]

Elucidation of in vitro chlorinated tyrosine adducts in blood plasma as selective biomarkers of chlorine exposure

Mirjam de Bruin-Hoegée[†,‡,*], Irene M. van Damme[†], Tomas van Groningen[‡], Debora van der Riet-van Oeveren[‡], Daan Noort[‡], Arian C. van Asten[†,§]

[†] van 't Hoff Institute for Molecular Sciences, Faculty of Science, University of Amsterdam, P.O. Box 94157, 1090GD Amsterdam, The Netherlands

[‡] TNO Defence, Safety and Security, Dep. CBRN Protection, Lange Kleiweg 137, 2288GJ Rijswijk, The Netherlands

[§] CLHC, Amsterdam Center for Forensic Science and Medicine, University of Amsterdam, P.O. Box 94157, 1090GD Amsterdam, The Netherlands

*Corresponding author. *Email address:* mirjam.debruin@tno.nl (M. de Bruin-Hoegée)

Supporting information

Contents

- S1. Method optimization and validation
- Figure S1. Calibration curves Cl-Tyr and di-Cl-Tyr
- Figure S2. Heatmap of peptides identified with LC-HRMS/MS after pepsin digestion
- Table S1. Extent of protein chlorination after pepsin digestion
- Table S2. Extent of protein chlorination after trypsin digestion
- Table S3. Identified biomarkers for chlorine exposure after trypsin and pepsin digestion.
- Figure S3. Protein coverage of human serum albumin and haptoglobin
- Figure S4. Extracted ion chromatograms of YLYEIAR
- S2. Isotope ratios
- Table S4. Theoretical compared to measured isotope values
- Table S5. Fragmentation pattern of peptide Y(Cl)LYEIAR
- Figure S5. MS/MS spectrum of parent ion K.Y(Cl)LY(Cl)EIAR
- Table S6. Fragmentation pattern of peptide Y(Cl)LY(Cl)EIAR
- Figure S6. MS/MS spectrum of parent ion K.Y(Cl)LY(Cl_2)EIAR
- Table S7. Fragmentation pattern of peptide Y(Cl)LY(Cl_2)EIAR
- Figure S7. Extracted ion chromatograms of HYEGSTVPEKK
- Table S8. Fragmentation pattern of peptide HY(Cl)EGSTVPEKK
- Figure S8. MS/MS spectrum of parent ion HY(Cl_2)EGSTVPEKK.
- Table S9. Fragmentation pattern of peptide HY(Cl_2)EGSTVPEKK

S1. Method optimization and validation

The LC-MS/MS method was optimized for Cl-Tyr (t_r: 6.15 min) and di-Cl-Tyr (t_r: 6.57 min) with $^{13}C_6$-3-chloro-L-tyrosine (t_r: 6.14 min) as internal standard. Linear calibration curves were obtained in the range of 1-100 ng/mL with R^2 = 0.9992-0.9999 (Figure S1). The mean values for the quality controls were within 20% at 1 ng/mL and within 6% relative standard deviation at both 10 ng/mL and 100 ng/mL (n = 10, for each concentration). The stability of the analytes was assessed over two weeks and the concentration was within 15% of the nominal value. The sample preparation efficiency of the pronase digest, determined by spiking known concentrations of analyte into human plasma, were respectively 59% ± 9% for Cl-Tyr and 56% ± 15% for di-Cl-Tyr. The ionization efficiency was slightly improved by the addition of 0.5% formic acid solution of 10 v% in water.

In addition, the influence of the plasma surface area on the effect of the chlorine exposure was examined. It was found that increasing the surface area with a factor of approximately 10 by using the laboratory glass bottle setup instead of a 15 mL polypropylene Corning tube resulted in a 5-7-fold increase in detected Cl-Tyr level in plasma (n = 3). Furthermore, it was found that increasing the exposure time from 2 hours to 48 hours did not significantly increase the degree of chlorination (concentrations were within the error range of ±1 stdv.). This was expected because blood plasma rapidly consumes chlorine. Finally, prolonging the pronase digestion duration beyond 48 hours did not significantly affect the detected levels of Cl-Tyr and di-Cl-Tyr.

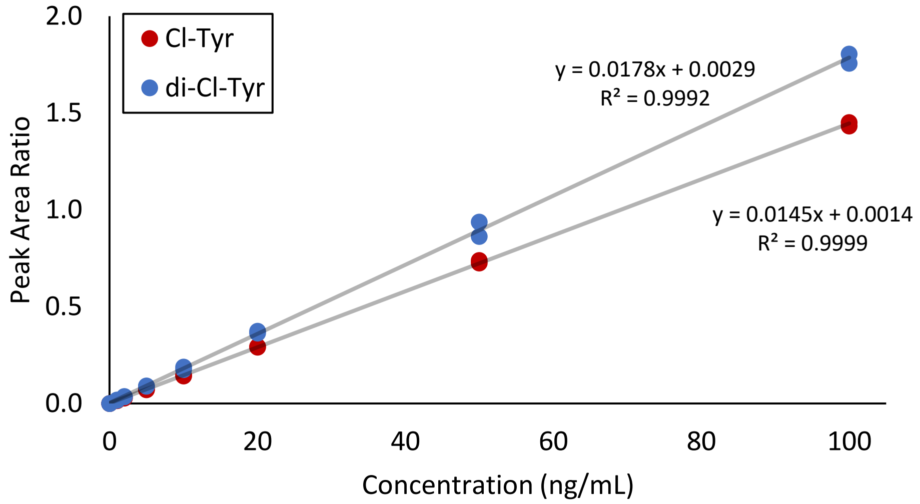

Figure S1. Calibration curves of 3-chlorotyrosine (Cl-Tyr) and 3,5-dichlorotyrosine (di-Cl-Tyr) with internal standard $^{13}C_6$-3-chloro-L-tyrosine, detected by LC-MS/MS.

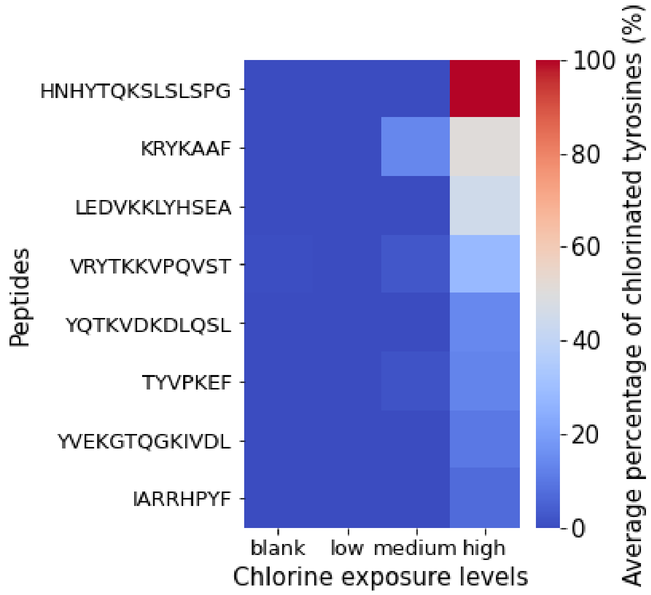

Figure S2. Heatmap of peptides identified with LC-HRMS/MS after pepsin digestion of human blood plasma, with corresponding average percentage of chlorinated tyrosines for non-exposed, low, medium, and high chlorine exposure.

Table S1. Extent of protein chlorination for various chlorine exposure levels after digestion by pepsin (n = 3, ±SD).

**Chlorine exposure level**	**Average percentage of chlorinated tyrosines (%)**
Blank	1.2 ± 0.4
Low	0.7 ± 0.6
Medium	3.2 ± 1.5
High	14 ± 9

Table S2. Extent of overall protein chlorination for various chlorine exposure levels after digestion by trypsin (n = 3, ±SD).

**Chlorine exposure level**	**Average percentage of chlorinated tyrosines (%)**
Blank	0.2 ± 0.3
Low	0.5 ± 0.5
Medium	2.3 ± 1.8
High	16.2 ± 2.1

Table S3. Identified biomarkers for chlorine exposure after trypsin and pepsin digestion.

Long peptide	Enzyme	Accession	Protein	Mass	t_R (min)	Cl (#)[a]	N (#)[b]
AHY(Cl)GGFTVQNEANK	Trypsin	P02675| FIBB_HUMAN	Fibrinogen beta chain	1568.7	18.5	1	2
AIGY(Cl)LNTGYQR	Trypsin	P01023| A2MG_HUMAN	Alpha-2-macroglobulin	1288.6	20.2	1	1
ALSHAVNNY(Cl)HK	Trypsin	P04114| APOB_HUMAN	Apolipoprotein B-100	1286.6	15.3	1	2
ASAGLLGAHAAAITAY(Cl)	Trypsin	P0C0L4| CO4A_HUMAN	Complement C4-A	1490.7	21.6	1	1
ATVLNY(Cl)LPK	Trypsin	P01023| A2MG_HUMAN	Alpha-2-macroglobulin	1051.5	21.9	1	3[c]
AVRPGY(Cl)PK	Trypsin	n.a.	n.a.	920.5	15.9	1	4[c]
DDLY(Cl)VSDAFHK	Trypsin	P01008| ANT3_HUMAN	Antithrombin-III	1342.6	20.4	1	3
FSVVY(Cl)AK	Trypsin	P02765| FETUA_HUMAN	Alpha-2-HS-glycoprotein	846.4	20.3	1	3
GEVPPRY(Cl)PR	Trypsin	P02790| HEMO_HUMAN	Hemopexin	1103.5	16.6	1	1
GGSTSY(Cl)GTGSETESPR	Trypsin	P02671| FIBA_HUMAN	Fibrinogen alpha chain	1605.6	16.3	1	2
GLSVY(Cl)ADKPETTK	Trypsin	P02763| A1AG1_HUMAN	Alpha-1-acid glycoprotein 1	1441.7	18.2	1	1
GVALHRPDVY(Cl).LLPPAR	Trypsin	P01871| IGHM_HUMAN	Immunoglobulin heavy constant mu	1159.6	19.1	1	2
GY(Cl)TQQLAFR	Trypsin	P01024| CO3_HUMAN	Complement C3	1116.5	20.5	1	2
AP.HGPGLIY(Cl)R	Trypsin	P02765| FETUA_HUMAN	Alpha-2-HS-glycoprotein	1113.5	19.2	1	1
HNHY(Cl)TQKSLSLSPG	Pepsin	P0DOX5| IGG1_HUMAN	Immunoglobulin gamma-1 heavy chain	1601.7	17.4	1	2
R.HPDY(Cl)SVVL.LLR	Trypsin	P02768| ALBU_HUMAN	Albumin	1500.8	21.5	1	1
HQLY(Cl)IDETVNSNIPTNLR	Trypsin	P02675| FIBB_HUMAN	Fibrinogen beta chain	1307.6	14.9	1	2
HY(Cl)EGSTVPEK.K	Trypsin	P00738| HPT_HUMAN	Haptoglobin	1307.6	14.9	1	5
HY(Cl2)EGSTVPEK.K	Trypsin	P00738| HPT_HUMAN	Haptoglobin	1341.6	15.1	2	3
YE.IARRHPY(Cl)F.Y(Cl)APEL	Pepsin	P02768| ALBU_HUMAN	Albumin	1360.6	20.8	2	1
L.IQPDSSVKPY(Cl)R	Trypsin	P02675| FIBB_HUMAN	Fibrinogen beta chain	1435.7	18.6	1	2

IY(Cl)GNQDTSSQLK	Trypsin	P19823| ITIH2_HUMAN	Inter-alpha-trypsin inhibitor heavy chain H2	1386.6	17.9	1	1
FA.KRY(Cl2)KAAF	Pepsin	P02768| ALBU_HUMAN	Albumin	1168.5	19.5	1	2[c]
FFA.KRY(Cl)KAAF	Pepsin	P02768| ALBU_HUMAN	Albumin	1281.6	20.6	1	4[c]
LEDVKKLY(Cl)HSEA	Pepsin	P01009| A1AT_HUMAN	Alpha-1-antitrypsin	1464.7	18.3	1	2
KQL.INDY(Cl)V.EK	Trypsin	P01011| AACT_HUMAN	Alpha-1-antichymotrypsin	1041.5	18.7	1	3[c]
LGEVNTY(Cl)AGDLQK	Trypsin	P06727| APOA4_HUMAN	Apolipoprotein A-IV	1440.7	20.0	1	2
LLIY(Cl)GASTR	Trypsin	n.a.	n.a.	1026.5	21.0	1	1
NSLFEY(Cl)QK	Trypsin	P02671| FIBA_HUMAN	Fibrinogen alpha chain	1061.5	20.6	1	3
EEAPSLR.PAPPPISGGGY(Cl)R	Trypsin	P02675| FIBB_HUMAN	Fibrinogen beta chain	1984.0	19.6	1	3
SY(Cl)STTAVVTNPK	Trypsin	P02766| TTHY_HUMAN	Transthyretin	1300.6	18.1	1	2
TAQEGDHGSHVY(Cl).TK	Trypsin	P01023| A2MG_HUMAN	Alpha-2-macroglobulin	1562.7	14.8	1	3
TY(Cl)ETTLEK	Trypsin	P02768| ALBU_HUMAN	Albumin	1017.4	18.2	1	6
SALE.VDETY(Cl)VPK	Trypsin	P02768| ALBU_HUMAN	Albumin	1383.6	20.6	1	1
EVDE.TY(Cl)VPKE.F	Pepsin	P02768| ALBU_HUMAN	Albumin	1388.6	21.7	1	1
VGPEADKY(Cl)R	Trypsin	P02679| FIBG_HUMAN	Fibrinogen gamma chain	1067.5	16.3	1	1
IV.VRY(Cl)TKKVPQVST.PTL	Pepsin	P02768| ALBU_HUMAN	Albumin	1438.8	16.3	1	7[c]
IV.VRY(Cl2)TKKVPQVST.PTL	Pepsin	P02768| ALBU_HUMAN	Albumin	1897.0	20.5	1	4[c]
WY(Cl)VDGVEVH.NAK	Trypsin	P01857| IGHG1_HUMAN	Immunoglobulin heavy constant gamma 1	1449.6	16.0	1	4[c]
Y(Cl)AATSQVLLPSK	Trypsin	P01871| IGHM_HUMAN	Immunoglobulin heavy constant mu	1310.7	20.2	1	2
F.Y(Cl)APELLFFAK	Trypsin	P02768| ALBU_HUMAN	Albumin	1378.7	22.2	1	2
Y(Cl)EKPGSPPR	Trypsin	P02751| FINC_HUMAN	Fibronectin	1063.5	15.3	1	1
Y(Cl)GAATFTR	Trypsin	P01023| A2MG_HUMAN	Alpha-2-macroglobulin	919.4	18.8	1	2
SI.Y(Cl)KPGQTVK	Trypsin	P01023| A2MG_HUMAN	Alpha-2-macroglobulin	1153.6	17.4	1	4

YLQEIY(Cl)NSNNQK	Trypsin	P02679|FIBG_HUMAN	Fibrinogen gamma chain	1546.7	19.6	1	1
K.Y(Cl)LY(Cl)EIAR	Trypsin	P02768|ALBU_HUMAN	Albumin	994.4	21.6	2	3
KK.Y(Cl)LYEIAR	Trypsin	P02768|ALBU_HUMAN	Albumin	960.4	20.8	1	4
Y(Cl)LY(Cl2)EIAR	Trypsin	P02768|ALBU_HUMAN	Albumin	1028.4	21.9	3	1
Y(Cl)QQHPGKAPK	Trypsin	P01704|LV214_HUMAN	Immunoglobulin lambda variable 2-14	1186.6	14.3	1	1
Y(Cl2)QQKPGKAPK	Trypsin	A0A0C4DH67|KV108_HUMAN	Immunoglobulin kappa variable 1-8	1594.8	15.2	2	1
Y(Cl)QQKPGQAPR	Trypsin	P01619|KV320_HUMAN	Immunoglobulin kappa variable 3-20	1205.6	14.8	1	5
Y(Cl)QTKVDKDLQSL	Pepsin	P02679|FIBG_HUMAN	Fibrinogen gamma chain	1470.7	20.0	1	1
Y(Cl)TFELSR	Trypsin	P02774|VTDB_HUMAN	Vitamin D-binding protein	948.4	20.9	1	2
Y(Cl)VEKGTQGKIVDL	Pepsin	P01009|A1AT_HUMAN	Alpha-1-antitrypsin	1482.7	20.0	1	1
Y(Cl)VGGQEHFAH	Trypsin	P02763|A1AG1_HUMAN	Alpha-1-acid glycoprotein 1	1177.5	16.8	1	1

[a]Number of attached chlorine atoms (Cl)

[b]Peptides detected in given number of repetitions (N)

[c]Not detected in all high chlorine exposure samples

A)

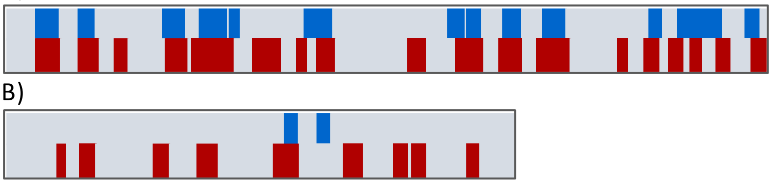

Figure S3. Protein coverage, with sequences identified after pepsin digestion (blue) and trypsin digestion (red). A) Human serum albumin (56% coverage), B) Haptoglobin (33% coverage).

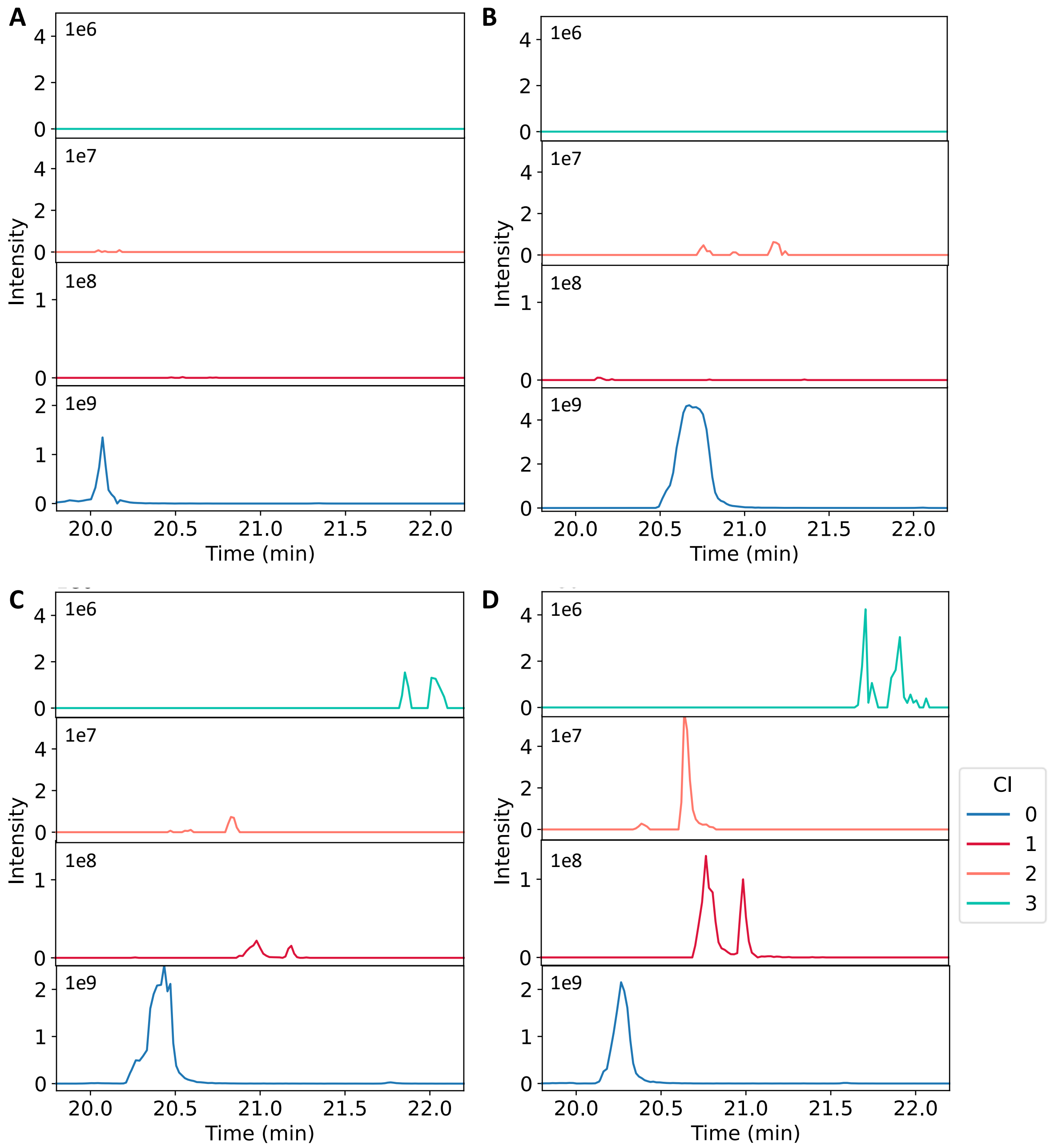

Figure S4. Extracted ion chromatograms of plasma exposed to various chlorine exposure concentrations analyzed by LC-HRMS/MS (Acclaim PepMap C18 column, mobile phase: H_2O (A) and ACN (B) with 0.1% FA), with YLYEIAR at m/z 464.25 and t_R = 20.1-20.7 min (blue), Y(Cl)LYEIAR and YLY(Cl)EIAR at m/z 481.23 and t_R = 20.8-21.0 and 21.0-21.2 min (red), K.Y(Cl)LY(Cl)EIAR at m/z 562.26 and t_R = 20.6-20.8 min (orange), and Y(Cl)LY(Cl_2)EIAR and Y(Cl_2)LY(Cl)EIAR at m/z 515.19 and t_R = 21.7-21.9 and 21.9-22.0 min (green). A) Blank, B) Low exposure, C) Medium exposure, D) High exposure.

S2. Isotope ratios

In the full scan MS spectrum of this doubly charged peptide, a distinct chlorine pattern is visible for single, double and triple chlorination (Figure 6B-D). The unchlorinated peptide showed a single peak as expected (Figure 6A). Because the ^{35}Cl isotope has a natural occurrence of 76% and the ^{37}Cl isotope of 24%, the isotope ratio for the chlorinated peptide can be calculated. When the influence of other isotopes, such as carbon, is also considered, the isotope ratio of a mono-, di- and tri-chlorinated peptide is 2:1 (Figure 6B), 5:4:1 (Figure 6C) and 11:12:5:1 (Figure 6D), respectively.[49] In Figure 6C, this last peak for the tri-chlorinated peptide was expected at a m/z 518.19, but it was not visible presumably due to its low intensity.

Table S4. Theoretical compared to measured isotope values in full scan MS spectrum analyzed by LC-HRMS/MS of doubly charged chlorinated precursor Y*LY*EIAR.

Chlorination	Theoretical isotope value	Measured isotope value
0	1	1
1	2:1	2:1
2	5:4:1	5:4:1
3	11:12:5:1	11:13:4:0

Table S5. Fragmentation pattern of peptide Y(Cl)LYEIAR with y and b fragments.

#	b (m/z)	Peptide	Y (m/z)	#
1	198.03	Y(+33.96)		7
2	311.12	L	764.43	6
3	474.18	Y	651.35	5
4	603.22	E	488.28	4
5	716.31	I	359.24	3
6	787.34	A	246.16	2
7		R	175.12	1

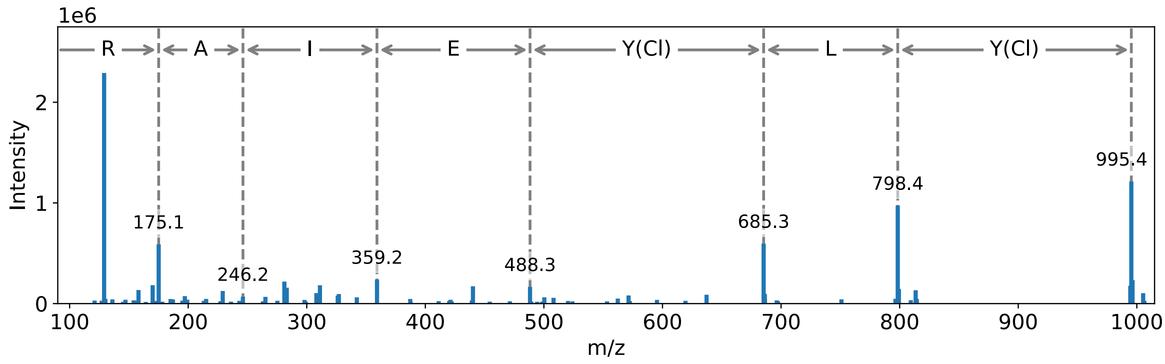

Figure S5. MS/MS spectrum of parent ion K.Y(Cl)LY(Cl)EIAR with m/z 562.262 at an t_R = 20.64 min., detected in the trypsin digest of a plasma sample exposed to 70 and 350 mmol/L chlorine gas. The m/z of the y-fragments and corresponding amino acids are shown.

Table S6. Fragmentation pattern of peptide Y(Cl)LY(Cl)EIAR with y and b fragments.

#	b (m/z)	Peptide	Y (m/z)	#
1	129.10	K		8
2	326.13	Y(+33.96)	995.41	7
3	439.21	L	798.39	6
4	636.24	Y(+33.96)	685.31	5
5	765.28	E	488.28	4
6	878.36	I	359.24	3
7	949.40	A	246.16	2
8		R	175.12	1

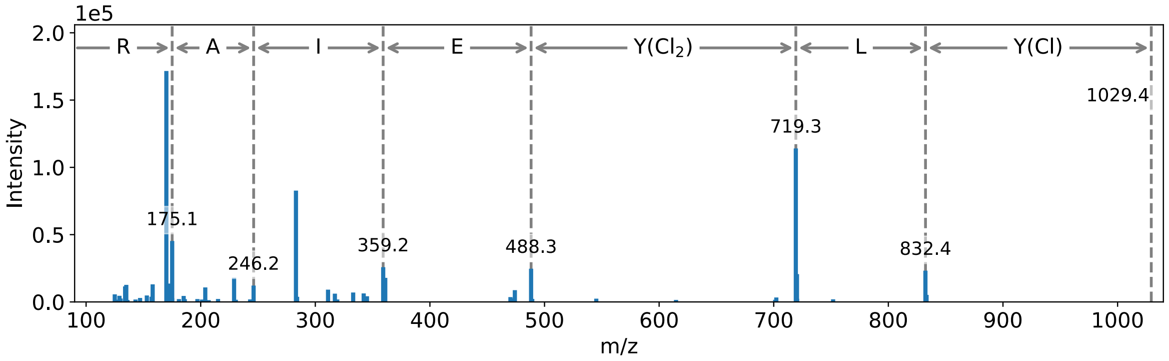

Figure S6. MS/MS spectrum of parent ion Y(Cl)LY(Cl_2)EIAR with m/z 515.194 at an t_R = 21.91 min., detected in the trypsin digest of a plasma sample exposed to 350 mmol/L chlorine gas. The m/z of the y-fragments and corresponding amino acids are shown.

Table S7. Fragmentation pattern of peptide Y(Cl)LY(Cl_2)EIAR with y and b fragments.

#	b (m/z)	Peptide	Y (m/z)	#
1	198.03	Y(+33.96)	1029.4	7
2	311.12	L	832.36	6
3	542.10	Y(+67.92)	719.27	5
4	671.14	E	488.28	4
5	784.23	I	359.24	3
6	855.27	A	246.16	2
7		R	175.12	1

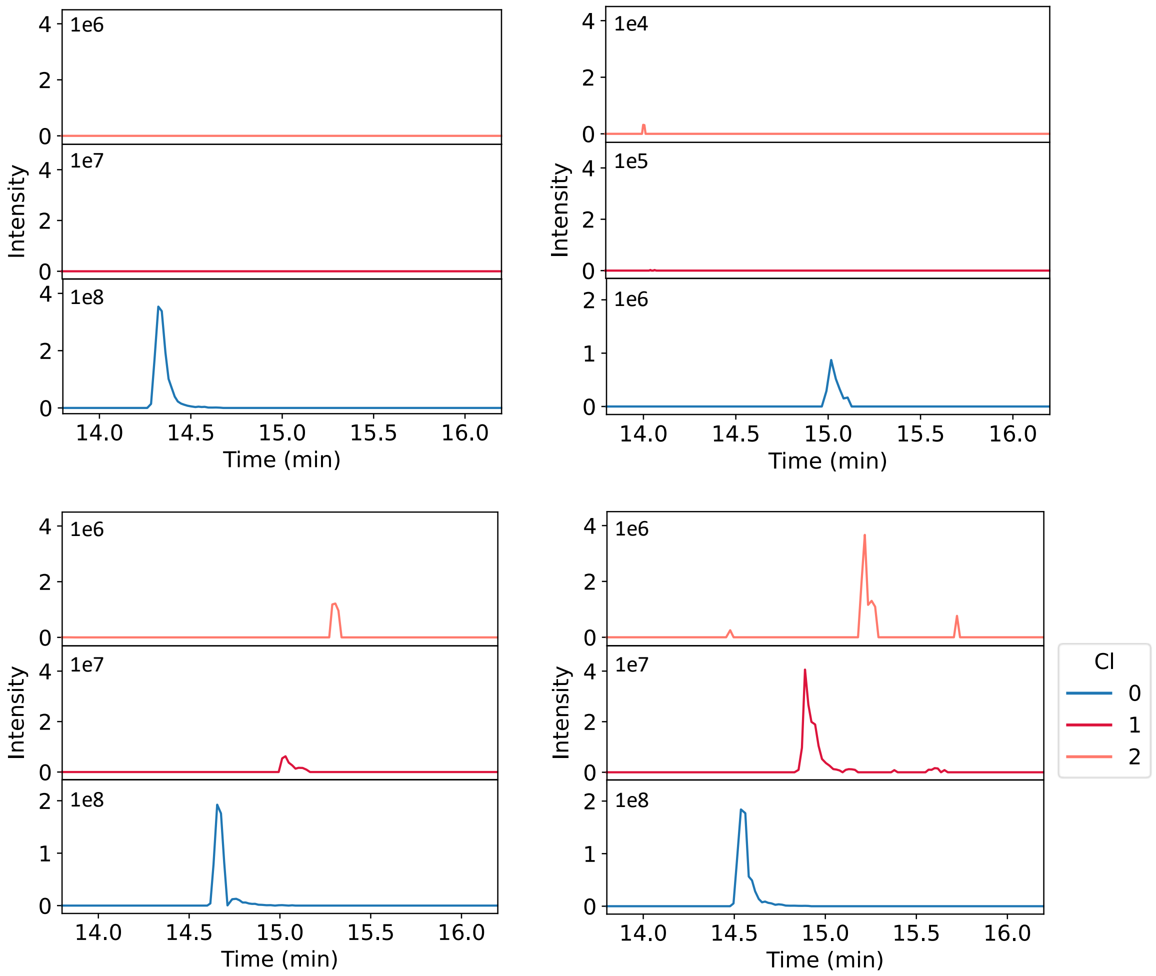

Figure S7. Extracted ion chromatograms of plasma exposed to various chlorine exposure concentrations analyzed by LC-HRMS/MS (Acclaim PepMap C18 column, mobile phase: H2O (A) and ACN (B) with 0.1% FA), with HYEGSTVPEKK at m/z 637.823 and t_R = 14.3-15.0 min (blue), HY(Cl)EGSTVPEKK at m/z 654.805 and t_R = 14.9-15.0 min (orange), and HY(Cl2)EGSTVPEKK at m/z 671.785 and t_R = 15.2-15.3 min (green). A) Blank, B) Low exposure, C) Medium exposure, D) High exposure.

Table S8. Fragmentation pattern of peptide HY(Cl)EGSTVPEKK with y and b fragments.

#	b	Peptide	y	#
1	138.07	H	1308.60	11
2	335.09	Y(+33.96)	1171.54	10
3	464.14	E	974.52	9
4	521.15	G	845.48	8
5	608.19	S	788.45	7
6	709.24	T	701.42	6
7	808.30	V	600.37	5
8	905.36	P	501.30	4
9	1034.40	E	404.25	3
10	1162.48	K	275.21	2
11		K	147.11	1

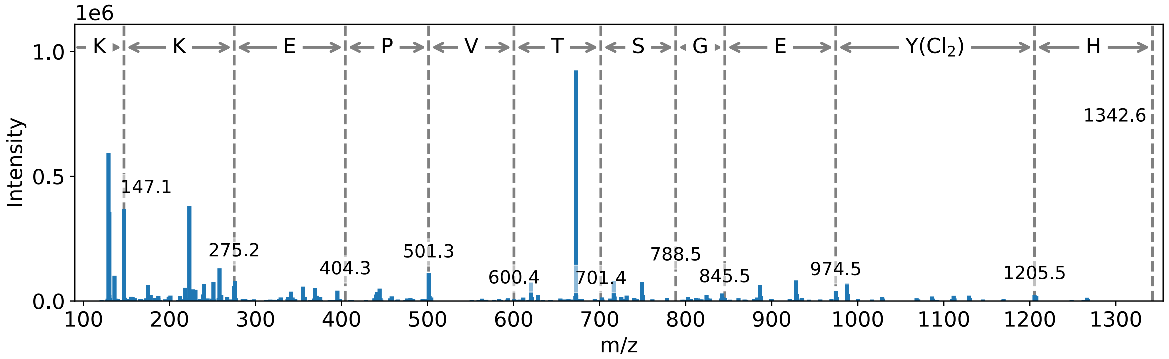

Figure S8. MS/MS spectrum of parent ion HY(Cl_2)EGSTVPEKK with m/z 672.648 at an t_R = 15.21 min., detected in the trypsin digest of a plasma sample exposed to 350 mmol/L chlorine gas. The m/z of the y-fragments and corresponding amino acids are shown.

Table S9. Fragmentation pattern of peptide HY(Cl_2)EGSTVPEKK with y and b fragments.

#	b	Peptide	y	#
1	138.07	H	1342.6	11
2	369.05	Y(+67.92)	1205.5	10
3	498.09	E	974.52	9
4	555.12	G	845.47	8
5	642.15	S	788.45	7
6	743.20	T	701.42	6
7	842.26	V	600.37	5
8	939.32	P	501.30	4
9	1068.4	E	404.25	3
10	1196.5	K	275.21	2
11		K	147.11	1
